# Deconvolution of the Voltammetric Features of a Pt(100) Single-Crystal Electrode

**DOI:** 10.1021/acs.jpclett.4c01056

**Published:** 2024-04-30

**Authors:** Xiaoting Chen, Kasinath Ojha, Marc T. M. Koper

**Affiliations:** ‡ School of Materials Science and Engineering, Beijing Institute of Technology, Beijing 100081, P. R. China; + Leiden Institute of Chemistry, Leiden University, P. O. Box 9502, 2300 RA, Leiden, The Netherlands

## Abstract

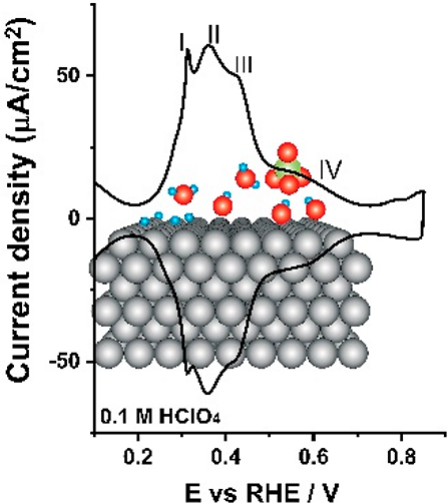

The Pt(100) single-crystal electrode shows four voltammetric features in acid electrolytes, but the precise corresponding surface phenomena remain unresolved. Herein, a deconvolution of the classical “hydrogen region” from the “hydroxyl and anion region” is attempted by the comparison of voltammetric behavior of Pt(100) and G_ML_Pt(100) electrodes. A systematic study performed on Pt(s)-[*n*(100) × (111)] and Pt(s)-[*n*(100) × (110)] electrodes reveals that the feature at *E*_PI_ = 0.30 V_RHE_ corresponds to pure hydrogen adsorption taking place at (111) step sites vicinal to (100) domains, while the peak at *E*_PII_ = 0.36 V_RHE_ actually involves hydroxyl replacing hydrogen at (100) domains. An analysis examined for H_2_SO_4_, HClO_4_, CH_3_SO_3_H, and HF demonstrates that the specific (H)SO_4_^–^ adsorption commences at *E*_PIII_ = 0.40 V_RHE_ and effectively suppresses the formation of hydroxyl at the (100) terrace at higher potentials 0.40 < *E*_PIV_ < 0.75 V_RHE_. Non-specifically adsorbing anions (ClO_4_^−^, CH_3_SO_3_^−^ and F^−^) would only interact with the hydroxyl phase formed on the Pt(100) terrace in both potential regions.

Platinum is regularly employed as an electro-catalyst for the conversion cycles of hydrogen, oxygen, and nitrogen species, where it catalyzes the hydrogen evolution/oxidation reaction (HER/HOR), the oxygen reduction reaction (ORR), the nitrate reduction, and the ammonia oxidation.^1−3^ Crystal facets govern the energy landscape of reactions at metal surfaces and thus the chemical interactions and binding strength of reaction intermediates on the Pt electrode surface. Surface structure is therefore of paramount importance for a large number of electrochemical reactions: the activity, selectivity, and mechanism of these reactions show remarkable structural dependence.^1,4−6^ Well-defined platinum surfaces (i.e., Pt single-crystal electrodes) are indispensable tools for the understanding of the electrochemical reactivity of catalytic reactions at the atomic/molecular level. The pioneering work of the flame annealing technique in 1980^7^ and its application to the electrochemical characterization of platinum single-crystal electrodes leads to invaluable insights into platinum surface electrochemistry relevant to energy storage and catalysis. It has been well accepted that the surface crystallographic orientation and the electrolyte composition result in a unique voltammetric signature involving the underpotential deposition of hydrogen, H_upd_, and the adsorption of hydroxyl and/or anions, consequently providing a “fingerprint” of a high-quality platinum single-crystal surface under electrochemical conditions.

Among basal planes, a great deal of knowledge has been gathered about the species responsible for the well-defined and reproducible voltammetric profile of the hexagonally close-packed Pt(111) electrode. Figure 1a shows three distinguishable regions within the potential window of ca. 0.0 to 0.90 V_RHE_ for the blank cyclic voltammogram of the Pt(111) electrode^8−14^ in a typical nonspecifically adsorbing electrolyte (i.e., perchloric acid): a hydrogen underpotential adsorption/desorption region (0.05 < *E* < 0.40 V_RHE_), a double-layer region (0.40 < *E* < 0.60 V_RHE_), and a hydroxyl adsorption/desorption region (0.60 < *E* < 0.90 V_RHE_). Although Pt(100) is widely recognized as the state-of-the-art electrocatalyst for nitrite reduction^15,16^ and ammonia oxidation,^17^ the electrochemistry of the well-defined Pt(100) surface is not as well understood as for the Pt(111) surface. This arises from the effect that the surface structure, that is, the large two-dimensional (100) domains, is much more sensitive to the conditions of sample cooling and has a higher instability (i.e., partial reconstruction introduced during a transfer operation) after the flame annealing technique than (111) domains.^18−20^Figure 1b depicts the main characteristics of the voltammetry of a well-ordered Pt(100) in perchloric acid: the voltammogram is characterized by four well-delineated peaks or features (*E*_PI_ = 0.30 V_RHE_, *E*_PII_ = 0.36 V_RHE_, *E*_PIII_ = 0.40 V_RHE_, and 0.40 < *E*_PIV_ < 0.75 V_RHE_), which have been ascribed to a convoluted “hydrogen desorption and hydroxyl/anion adsorption” region.^14,21−23^ Feliu et al. have proposed that the hydrogen and OH adsorption (partially) overlap on the Pt(100) electrode based on temperature-dependent thermodynamics experiments.^24^ Using in situ Fourier transform infrared (FTIR) spectroscopy, Hoshi and Nakamura reported the onset potential of OH_ads_ formation on the Pt(100) electrode at ca. 0.30 V in 0.1 M perchloric acid.^23^ Spectroscopic work revealed contributions of strongly specifically adsorbing anions, namely, acetate^25^ and (bi)sulfate anion,^26,27^ at potentials higher than that of ca. 0.37 V in NaAc and sulfuric acid. CO charge replacement locates the pztc (potential of zero total charge) of Pt(100) at 0.41 V_RHE_ (i.e., in the middle of the broad voltammetric profile).^25^ This would agree with the idea of (at least) two adsorbates adsorbing within this region, leading to a zero total charge balance somewhere in the middle.

**Figure 1:**
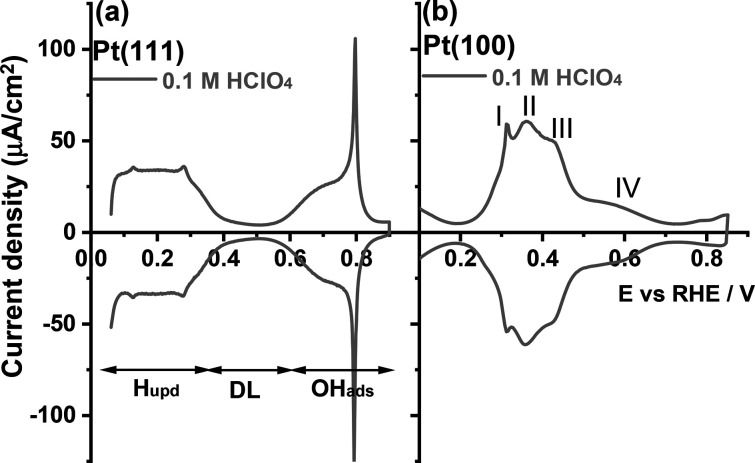
Cyclic voltammograms of (a) Pt(111) and (b) Pt(100) electrodes recorded in 0.1 M HClO_4_. Scan rate: 50 mV s^–1^.

The objective of this work is to update our understanding of the elementary surface phenomena corresponding to the blank cyclic voltammogram of a Pt(100) single-crystal electrode. We show that a deconvolution of the “hydrogen desorption and hydroxyl/anion adsorption” region on Pt(100) can be achieved by the deposition of a single monolayer of graphene. We then perform systematic studies on the electrochemical behavior of the Pt(100) vicinal surfaces and assign the deconvoluted hydrogen desorption and hydroxyl/anion adsorption peaks appearing in the voltammetric profile to specific sites. These results improve our fundamental understanding of hydrogen, hydroxyl, and anion adsorption on a well-defined single-crystal Pt(100) surface, which will be important for interpreting and tuning the catalytic activity of platinum-based electrochemical interfaces.

Pt(111)^28,29^ and Pt(100)^30,31^ single-crystal electrodes can be modified with a single monolayer of graphene by ambient-pressure chemical vapor deposition. *In situ* low-energy electron microscopy (LEED) and STM measurements lend support to the notion of macroscopic monolayer graphene domains, and simulations give a separation of 2.4 Å < *d* < 3.7 Å across the graphene–Pt interface^32,33^ (as indicated in Figure 2a). As defects in the monolayer graphene are selectively permeable to H^+^ ions in the electrolyte, allowing only H^+^ ions to enter the confined layer between graphene (Figure 2a) and the topmost atomic layer of the Pt electrode,^34−36^ it offers a powerful modification to distinguish different electrochemical adsorption reactions (i.e., hydrogen adsorption/desorption) from other processes (i.e., hydroxyl and any other anion adsorption/desorption). Figure 2b first shows cyclic voltammograms for the Pt(100) electrode in 0.1 M HClO_4_ and 0.1 M H_2_SO_4_ electrolytes (i.e., the typical nonspecifically adsorbing and strongly specifically adsorbing electrolytes, respectively).^21^ The cyclic voltammograms for Pt(100) in 0.1 M H_2_SO_4_ and 0.1 M HClO_4_ show typical features as discussed in the introduction. Notably, the voltammograms in HClO_4_ and H_2_SO_4_ overlap perfectly at potentials below *E*_PII_ = 0.36 V, indicating that anion adsorption, perchlorate and (bi)sulfate, does not participate in these low-potential adsorption states.

**Figure 2:**
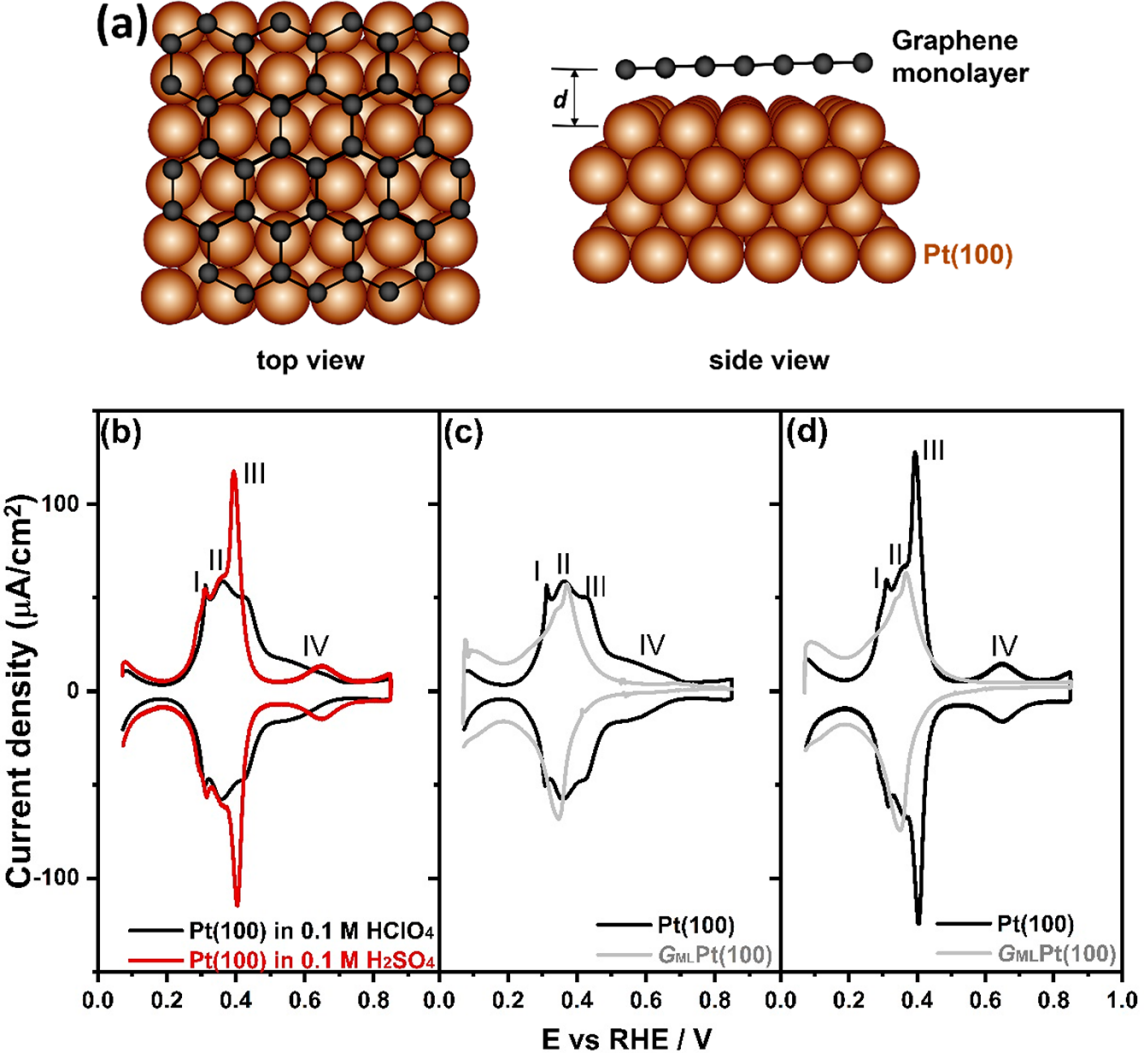
(a) Top and side views of the structure of the graphene monolayer on the Pt(100) single-crystal electrode (G_ML_Pt(100)). (b) Cyclic voltammograms of the Pt(100) electrode recorded in 0.1 M HClO_4_ (black line) and 0.1 M H_2_SO_4_ (red line). Cyclic voltammograms of the G_ML_Pt(100) electrode recorded in (c) 0.1 M HClO_4_ and (d) 0.1 M H_2_SO_4_ electrolytes. Scan rate: 50 mV s^–1^.

Figure 2c,d shows cyclic voltammograms for Pt(100) and G_ML_Pt(100) (a graphene monolayer-coated Pt(100) electrode) in 0.1 M HClO_4_ and 0.1 M H_2_SO_4_ electrolytes, respectively. The important observation in Figure 2c,d is that the voltammetric peaks attributed to the respective anion adsorption process (P_III_ and P_IV_) vanish on the G_ML_Pt(100) electrode. Another effect of the introduction of graphene is the concomitant increase in current density in the lower potential region of the voltammogram between 0.20 V_RHE_ and the beginning of hydrogen evolution. The feature in this low potential region has been assigned to hydrogen de/adsorption at step sites on the Pt(100) single-crystal electrode.^25^ Analogously, the voltammetric modification at a potential lower than 0.20 V_RHE_ in Figure 2c,d may imply the disruption of long-range (100) domains during graphene deposition and can also be qualitatively compared with the changes caused by introducing step sites to (100) domains (as will be further discussed in relation to Figure 3). It is also observed that the shape of the hydrogen de/adsorption peak at 0.30 V_RHE_ on the G_ML_Pt(100) electrode (gray curve) appears slightly changed compared to the bare Pt(100) electrode (black curve), suggesting a change in the interactions between the adsorbed hydrogen atoms on Pt(100) due to the presence of graphene (a similar change has been observed for Pt(111)^35^). As a result, voltammetric peaks at potentials more negative than *E*_PII_ = 0.36 V_RHE_ could be assigned to a pure hydrogen adsorption/desorption process:
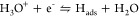
1

**Figure 3:**
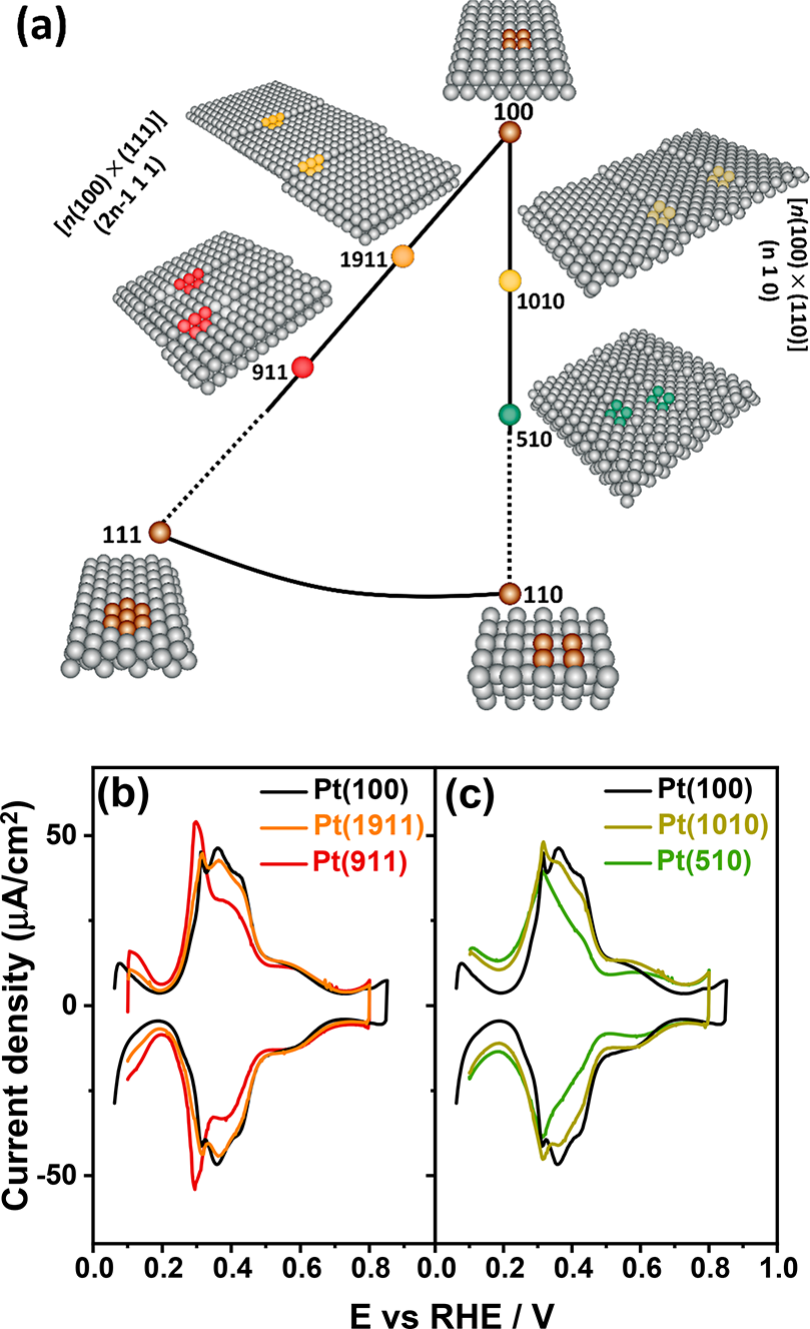
(a) Stereographic triangle for a face-centered cubic crystal with hard sphere models of some selected surfaces, indicating the main dimensions of the unit cell. Cyclic voltammograms of (b)Pt(100), Pt(19,1,1), and Pt(911) and (c) Pt(100), Pt(10,1,0), and Pt(510) surfaces in 0.1 M HClO_4_. Scan rate: 50 mV s^–1^.

Previous studies^18,25^ have proposed that the H_upd_ on the Pt(100) electrode (eq 1) has two different contributions: (i) a state at low potentials (0.05 < *E* < 0.36 V_RHE_) which is attributed to sites on the two-dimensional-ordered (100) terraces and (ii) specific voltametric peaks at *E*_PI_ = 0.30 V_RHE_ and *E*_PII_ = 0.36 V_RHE_ which would arise from hydrogen de/adsorption at step sites vicinal to (100) terraces. In order to understand the exact nature of the adsorption sites on Pt(100), a comparison can be made to the voltammograms of a series of stepped surfaces in the same crystallographic zone.

Figure 3b,c shows the cyclic voltammograms of Pt(s)-[*n*(100) × (111)]- and Pt(s)-[*n*(100) × (110)]-oriented electrodes, with (100) terrace widths of *n* = 10 and 5 atoms, separated by monatomic (111) and (100) steps (as indicated in Figure 3a), respectively. An increase in the step density of the (111) orientation increases the hydrogen adsorption/desorption states at peak I (*E*_PI_ = 0.30 V_RHE_) (Figure 3b), while the presence of (100)-oriented steps does not lead to a noticeable change in peak I (Figure 3c). Peak II decreases with increasing step density (or decreasing terrace width), and comparison to Figure 2 would indicate that this peak corresponds to hydrogen adsorption on the 2D (100) terraces. The lateral interactions between the hydrogen adsorbates on steps and terraces have different signs: those on the terraces being repulsive (a broad feature) and those on the steps being apparently attractive (a sharp feature). Peaks III and IV at higher potentials (*E*_PIII_ = 0.40 V_RHE_ and 0.40 < *E*_PIV_ < 0.70 V_RHE_) should involve hydroxyl and/or anion adsorption/desorption as they are absent in voltammograms of the graphene-modified Pt(100) in Figure 2. Peak III must involve (100) terrace sites since the charge under this peak decreases with an increasing density of steps. For peak IV, the situation is less pronounced with (100) terrace widths of *n* = 10, and for the narrower terrace widths of *n* = 5, it shows a visible decrease (Figure 3). In perchloric acid, the OH adsorption charge for 0.40 < *E*_PIV_ < 0.70 V_RHE_ decreases slightly as the step density increases up to *n* values around 6, with the decrease in the amount of charge related to OH adsorption being important for smaller *n*. A considerable diminution of the intensity of the adsorption processes at peak IV has been reported at the Pt(511) single-crystal electrode, a surface with (100) terrace widths of *n* = 3 atoms separated by monatomic (111) steps.^25^

Figure 4 shows cyclic voltammograms for the Pt(100) electrode in 0.001 M HClO_4_ (pH = 3) electrolytes in the absence and presence of cations. If a peak potential shifts with cation concentration or identity, then this can be taken as evidence that OH adsorption/desorption is involved.^9,37^ The peak potential of peak I (*E*_PI_ = 0.30 V_RHE_) is independent of both perchlorate concentration and cation concentration/identity, suggesting that this peak corresponds to H adsorption/desorption only. Surprisingly, the same holds for peak IV (0.40 < *E*_PIV_ < 0.70 V_RHE_), even though Figure 2b strongly indicates that this peak cannot involve the adsorption/desorption of only H. Figure 4a shows that with increasing concentration of the alkali metal cation (K^+^), peak II (*E*_PII_ = 0.36 V_RHE_) is shifted to more positive potential in comparison with the peak potential in HClO_4_. Figure 4b further illustrates that the shift is more pronounced for larger cations: for 0.01 M K^+^ (red line) and 0.01 M Cs^+^ (green line) containing electrolytes, the peak at *E*_PII_ = 0.36 V_RHE_ is shifted to 0.39 V_RHE_ and 0.41 V_RHE_, respectively. These results imply that the nature of the process (*E*_PII_ = 0.36 V_RHE_) is not due to just adsorption and desorption of hydrogen on (100) terrace sites but actually involves a replacement of hydrogen by hydroxyl, as the cation would cause a peak potential shift to a more positive value due to its destabilization of the “hydroxyl-cation” adlayer:
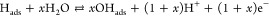
2

**Figure 4:**
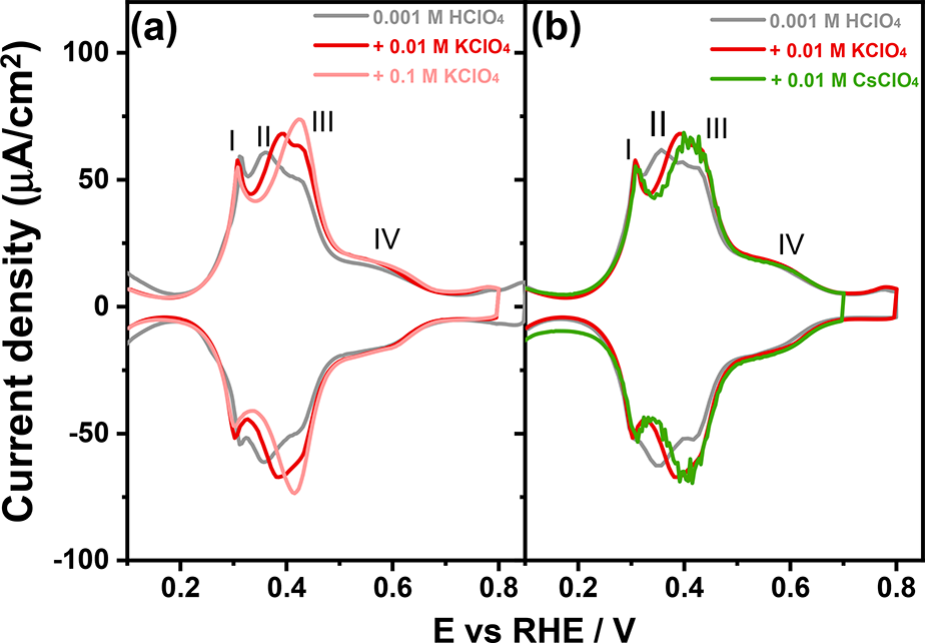
Cyclic voltammograms of Pt(100) in (a) 0.001 M HClO_4_ (pH = 3) solution without (gray line) and with 0.01 M KClO_4_ (red line) and 0.1 M KClO_4_ (pink line) solutions. (b) Cyclic voltammograms of Pt(100) in 0.001 M HClO_4_ (pH = 3) solution without (gray line) and with 0.01 M KClO_4_ (red line) and 0.01 M CsClO_4_ (green line) solutions. Scan rate: 50 mV s^–1^.

This effect of the cation on the peak potential is identical to the effect that we observed previously for the step-related “hydrogen” peaks on stepped Pt electrodes^9,37^ and Pd_ML_Pt(111) electrodes.^38^ Recently, Feliu et al.^39^ performed electrochemical shell-isolated nanoparticle-enhanced Raman spectroscopy (EC-SHINERS) and demonstrated the presence of adsorbed OH (after replacing adsorbed H) at Pt surface sites with a low coordination number at low potentials (i.e., in the traditional hydrogen region).

Here, the conclusion that peak II would involve OH_ads_ seems to contradiction the observation that peak II is still observed in the CV of the graphene-modified Pt(100). However, we earlier noted another anomaly, namely, that the current in the region below 0.2 V_RHE_ is much higher after graphene modification (Figure 2). Such a higher current is also observed for the unmodified stepped Pt surfaces (Figure 3) and therefore raises the question of whether the graphene modification changes the structure of the underlying Pt(100) surface. Studies from LEED observanons in UHV systems confirmed that a possible reconstruction of the Pt(100) surface was lifted after monolayer graphite deposition.^33^ On the other hand, a continuous sheet of graphene can be grown perfectly across Pt(100) domain boundaries, and angular shift/buckling of the graphene layer could be caused by the incorporation of pentagon–heptagon across step edges.^30^ While graphene modification of Pt(100) suppresses OH_ads_ adsorption and allows us to state that peaks III and IV involve OH_ads_, the presence of graphene also appears to change the underlying structure of the Pt(100) electrode; therefore, the situation is much less clear for peak II. Since this peak is sensitive to cations, we conclude that it must still include OH_ads_ adsorption. Therefore, we must be careful in presuming a one-to-one correspondence of the peaks on the Pt(100) to the G_ML_Pt(100) electrode.

Figure 5a shows the voltammograms of a Pt(100) electrode obtained with several anions, namely, 0.1 M HClO_4_, 0.1 M HF, 0.1 M CH_3_SO_3_H, and 0.1 M H_2_SO_4_ solution. The feature corresponding to the hydrogen adsorption/desorption at (111) step sites vicinal to (100) terraces (*E*_PII_ = 0.30 V_RHE_, eq 1) as well as peak II, presumably corresponding mainly to H adsorption/desorption on the (100) terraces, is insensitive to the nature of the anion. By contrast, Figure 5a shows that peak III (*E*_PIII_ = 0.40 V_RHE_) is sensitive to anion identity (and concentration, as shown in Figure 4) and the peak intensity increases in the order of ClO_4_ ≤ F ≤ CH_3_SO_3_H ≪(H)SO_4_, even if the effect of perchlorate, fluoride, and methanesulfonate is very small. It has been evidenced by spectro-electrochemical experiments that the bands corresponding to adsorbed (bi)sulfate on the Pt(100) surface start to appear at ca. 0.40 V_RHE_.^26^

**Figure 5:**
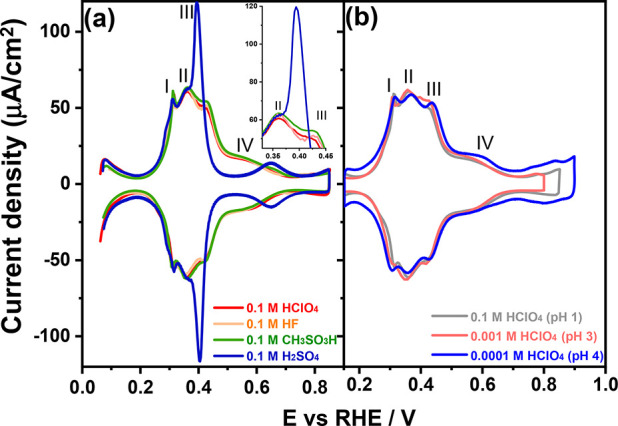
Cyclic voltammograms of Pt(100) in (a) 0.1 M HClO_4_, 0.1 M HF, 0.1 M CH_3_SO_3_H, and 0.1 M H_2_SO_4_ solution. The inset represents a magnification of the chosen potential of *E*_PII_ and *E*_PIII_. (b) Cyclic voltammograms of Pt(100) in 0.1 M HClO_4_ (pH = 1), 0.001 M HClO_4_ (pH = 3), and 0.0001 M HClO_4_ (pH = 4) solutions. Scan rate: 50 mV s^–1^.

A consistent explanation of this effect, which is quite similar to what we have observed on the Pd_ML_Pt(111) surface,^38^ is that the peak around *E*_PIII_ = 0.40 V_RHE_ involves either the (weak) specific adsorption of these anions or the interaction of nonspecifically adsorbing anions with the OH_ads_. Consistent with the latter interpretation is that Figure 5 also shows that the intensity of peak III grows with increasing concentration of nonspecifically adsorbing anion (perchlorate) concentration at a constant pH. There appears to be no variation in the onset nor any shift associated with the broad adsorption state on the (100) terrace (0.40 < *E*_PIV_ < 0.70 V_RHE_) in working electrolytes with nonspecifically adsorbing anions.

Figure 5a shows that the specific adsorption of the (bi)sulfate anion commences at *E*_PIII_ = 0.40 V_RHE_ (eq 3) and shifts peak IV to a higher potential region of 0.55 < *E*_PIV_ < 0.75 V_RHE_, with a noticeable decrease in coverage. Figure 5b further shows the evolution of the blank CV of Pt(100) in a typical nonspecifically adsorbing solution (i.e., perchloric acid) as a function of acid concentration. The OH_ads_ profiles on two-dimensional (100) domains between 0.40 and 0.70 V_RHE_ in 0.1 M HClO_4_ (pH = 1), 0.001 M HClO_4_ (pH = 3), and 0.0001 M HClO_4_ (pH = 4) solutions (as indicated in Figure 5b) suggest that OH_ads_ formation on the (100) terrace (0.40 < *E*_PIV_ < 0.70 V_RHE_, eq 4) is a bit more sensitive to the anion concentration than to the electrolyte pH. This would indicate that nonspecifically adsorbing anions (i.e., perchlorate, fluoride, and methanesulfonate) presumably interfere with the lateral interactions between adsorbed OH on large two-dimensional (100) domains, which has also been reported for large two-dimensional (111) domains (i.e., Pt(111) single-crystal electrodes).^40,41^

In this work, we have presented a characterization/deconvolution of the various superimposed electrochemical processes leading to various characteristic voltammetric peaks of a Pt(100) single-crystal electrode. The voltammetric fingerprint basically consists of four peaks I–IV. We showed the following:

(i) The current below peak I at *E*_PI_ = 0.30 V_RHE_ is due to adsorption and desorption of only hydrogen at (111) step sites vicinal to (100) large domains.
(ii) Peak II at *E*_PII_ = 0.36 V_RHE_ actually involves the replacement of hydrogen by hydroxyl at two-dimensional (100) domains. This hydroxyl adsorption at low potentials is quite sensitive to the nature of the electrolyte cation.
(iii) Peak III at *E*_PIII_ = 0.40 V_RHE_ involves either the exchange of OH_ads_ with a strong specifically adsorbed (bi)sulfate anion or interactions with nonspecifical adsorbing anions in the manner of perchlorate ≤ fluoride ≤ methanesulfonate.
(iv) The OH_ads_ on large (100) terraces yields a broader feature at 0.40 < *E*_PIV_ < 0.75 V_RHE_, and the considered nonspecifically adsorbing anions interfere (weakly) with the lateral interactions among OH_ads_. A strongly adsorbed anion such as (bi)sulfate suppresses the hydroxyl adsorption and shifts the OH_ads_ on Pt(100) terrace sites to a much more positive potential region, with an apparently lower coverage.

## Methods

*Materials and Chemicals*. Electrolytes were made from ultrapure water, high-purity reagents HClO_4_ (60%), H_2_SO_4_ (96%), CH_3_SO_3_H (>99.0%), and HF (40%) from Merck Suprapur, and KClO_4_ (99.99%) and CsClO_4_ (99.995%) from Aldrich Ultrapure. Before each experiment, the electrolytes were first purged with argon (Air Products, 5.7) for at least 30 min to remove air from the solution. Afterward, a flow of argon was carefully introduced into the atmosphere above the electrolyte.

*Electrodes and Electrochemical Experiments*. Cyclic voltammetry measurements were carried out in standard electrochemical cells by using a three-electrode assembly at room temperature. Experiments were performed in a fluorinated ethylene propylene (PEP, Nalgene) electrochemical cell for hydrofluoric acid, and a glass cell was used for the other electrolytes. All glassware was cleaned in an acidic solution of potassium permanganate overnight, followed by rinsing with an acidic solution of hydrogen peroxide, repetitive rinsing, and boiling with ultrapure water. Bead-type Pt(100), Pt(111), Pt(1911), Pt(911), Pt(1010), and Pt(510) single-crystal electrodes (around 3 mm in diameter) were used as working electrodes (MaTecK). The Pt single-crystal electrode was prepared by a repeated flame annealing technique according to the Clavilier method.^7^ After confirming a high-quality Pt(100) single-crystal electrode, a single layer of high-quality graphene was grown in an induction cell via chemical vapor deposition (CVD) in a mixture of ethylene, hydrogen, and argon using the procedure reported in Fu et al.^34^ and in our previous work.^35,36^ A coiled platinum wire was used as a counter electrode, and a reversible hydrogen electrode (RHE, Mini HydroFlex, Gaskatel) was employed as the reference electrode. All potentials are reported versus the RHE. The electrochemical measurements were performed with a single-crystal electrode in the hanging meniscus configuration. The potential was controlled with either an Autolab PGSTAT302N or a Biologic VSP-300 potentiostat. The current density shown here represents the measured current normalized to the geometric area of the working electrode.

## Data Availability

The data sets generated during and/or analyzed during the current study are available from the corresponding author on reasonable request.
